# Sugar Palm Fibre-Reinforced Polymer Composites: Influence of Chemical Treatments on Its Mechanical Properties

**DOI:** 10.3390/ma15113852

**Published:** 2022-05-27

**Authors:** Muhammad Rizal Muhammad Asyraf, Agusril Syamsir, Abu Bakar Mohd Supian, Fathoni Usman, Rushdan Ahmad Ilyas, Norizan Mohd Nurazzi, Mohd Nor Faiz Norrrahim, Muhammad Rizal Razman, Sharifah Zarina Syed Zakaria, Shubham Sharma, Zarina Itam, Mohamad Zakir Abd Rashid

**Affiliations:** 1 Institute of Energy Infrastructure, Universiti Tenaga Nasional, Jalan IKRAM-UNITEN, Kajang 43000, Selangor, Malaysia; mohd.supian@uniten.edu.my (A.B.M.S.); fathoni@uniten.edu.my (F.U.); 2 School of Mechanical Engineering, Faculty of Engineering, Universiti Teknologi Malaysia (UTM), Johor Bahru 81310, Johor, Malaysia; 3 Centre for Advanced Composite Materials (CACM), Faculty of Engineering, Universiti Teknologi Malaysia (UTM), Johor Bahru 81310, Johor, Malaysia; ahmadilyas@utm.my; 4 School of Chemical and Energy Engineering, Faculty of Engineering, Universiti Teknologi Malaysia (UTM), Johor Bahru 81310, Johor, Malaysia; 5 Bioresource Technology Division, School of Industrial Technology, Universiti Sains Malaysia (USM), Gelugor 11800, Pulau Pinang, Malaysia; mohd.nurazzi@gmail.com; 6 Research Centre for Chemical Defence, Universiti Pertahanan Nasional Malaysia (UPNM), Kem Perdana Sungai Besi, Kuala Lumpur 57000, Malaysia; faiz@upnm.edu.my; 7 Research Centre for Sustainability Science and Governance (SGK), Institute for Environment and Development (LESTARI), Universiti Kebangsaan Malaysia (UKM), Bangi 43600, Selangor, Malaysia; mrizal@ukm.edu.my; 8 Research Centre for Environmental, Economic and Social Sustainability (KASES), Institute for Environment and Development (LESTARI), Universiti Kebangsaan Malaysia (UKM), Bangi 43600, Selangor, Malaysia; szarina@ukm.edu.my; 9 Mechanical Engineering Department, University Center for Research & Development (UCRD), Chandigarh University, Mohali 140413, Punjab, India; shubham543sharma@gmail.com; 10 Department of Mechanical Engineering, IK Gujral Punjab Technical University, Kapurthala 144603, India; 11 Civil Engineering Department, Universiti Tenaga Nasional, Jalan IKRAM-UNITEN, Kajang 43000, Selangor, Malaysia; izarina@uniten.edu.my; 12 TNB Grid Division, Grid Solution Expertise (GSE), Bangunan Dua Sentral No. 8, Jalan Tun Sambanthan, Kuala Lumpur 50470, Malaysia; zakir.rashid@tnb.com.my

**Keywords:** sugar palm, lignocellulosic fibre, chemical treatments, thermosetting and thermoplastic, mechanical properties, natural fibre-composites

## Abstract

In the era of globalisation, decreasing synthetic resources, especially petroleum, have encouraged global communities to apply biomass waste as a substitute material for green technology development. The development of plastic products from lignocellulosic fibre-reinforced composites has been a hot topic among material scientists and engineers due to their abundance, sustainable in nature, and less toxic towards health. For the Malaysian scenario, sugar palm is a plant found in the wild and locally planted in certain areas in Malaysia and Indonesia. Generally, sugar palm can be harvested for traditional foods, fruits, starch sugar (*gula kabung*), and alcohol, whereas sugar palm fibre (SPF) is used in conventional products (brushes and brooms). Various researchers are working on the characterisation of fibre and its composites for engineering and packaging products. The main drawback of SPF is its hydrophilic behaviour, which leads to high moisture uptake and inhibits a good bond between the fibre and the matrix. Thus, a solution for this problem is by implementing chemical treatments on the fibre. From the literature review, no comprehensive review paper has been published on the influence of chemical treatment on the mechanical behaviour of SPF-reinforced polymer composites. Thus, the present review examines recent studies on the mechanical properties of sugar palm lignocellulosic fibres with various chemical treatments to evaluate their potential in structural applications.

## 1. Introduction

Recently, plant wastes, such as sugar palm fibre (SPF), coconut sheath, and kenaf stem, have been used in various applications due to their widespread availability and low costs [1]. Lignocellulosic fibres are implemented by industries in many sectors to grasp the idea of sustainability [2,3]. In general practice, these fibres are implemented together with a polymer matrix to form biocomposite products. Various lignocellulosic fibres, including flax [4], kenaf [5], pineapple leaf [6], and sugar palm [7] have been applied in many engineering applications. The increasing trend in the use of lignocellulosic fibre composites has shown that composite materials have received significant attention from the public [8]. This situation is beneficial to global communities in order to reduce the reliance and dependence on synthetic materials [9]. The main benefits of these polymer composites include availability and various advantages, eco-friendly to the environment, good thermal insulation properties, good formability, low cost, renewable, and sufficient energy requirements [10,11,12]. In terms of chemical composition, these types of fibres are mainly composed of cellulose as the structural building material reinforced in the lignin matrix [13]. Other components, such as hemicellulose, lignin, pectin, waxes, and water-soluble substances, express green plant characteristics. The components of lignocellulosic fibres may vary from one to another, depending on plant species, growth conditions, geographical locations, fibre extraction techniques, as well as the height and parts of the plant [14,15,16,17].

*Arenga pinnata* or SPF exhibits significant fibre strength and stiffness that can be applied for composite reinforcement. Generally, SPF is one of the widely available fibres that can be found in Malaysia, Indonesia, and other South Asian countries. SPF has also been widely used in various product development activities, including packaging, food container, furniture, helmets, and boats, indicating that this fibre contributes to the advancement of green technology [18,19,20,21,22]. The various SPF-reinforced polymer composites are beneficial due to their good mechanical performance, which is useful for structural and automobile sectors. Many engineers and scientists have demonstrated higher mechanical performance of SPF-reinforced polymer composites than that of other types of lignocellulosic fibre polymer composites; hence, lignocellulosic fibre-reinforced polymer composites are suitable for high-structural performance [23,24]. According to Misri et al. [25], SPF-reinforced polymer composites have been used to develop fishing boats, which exhibit good resistance to water permeability, as well as high mechanical strength. This shows that SPF has the potential to be embedded in automotive components [26,27], body armour [28], structures in transmission tower [29,30,31,32], and tissue engineering products [33].

Even though lignocellulosic fibres, such as SPF, possess various advantages, they exhibit hydrophilic characteristics with high water absorption [34]. This reduces their bonding wettability with hydrophobic polymer resin, leading to a lack of interfacial bonding between the fibre and the matrix. This condition may induce non-uniformity that limits the mechanical strength of the composites and consequently results in unfavourable properties [35,36,37,38]. In order to resolve this issue, SPF pretreatment is suggested by several researchers either via physical or chemical techniques [39,40]. The performance of the composites can be enhanced by altering the compatibility between fibre and polymer to form effective composites. Specifically, the treated lignocellulosic fibre has a rougher surface, which contributes to more activation areas for chemical bonding with matrix [41,42,43]. A good network of fibre/matrix subsequently provides superior composite strength and enhances the use of lignocellulosic fibre for structural, automobile, aerospace, aircraft, and household products [44,45,46,47,48,49].

Currently, numerous review articles elaborate on SPF and its composites with various characterisation techniques [50,51,52,53]. To the best knowledge of the authors, these reviews are still limited, inadequate, and slightly informative, especially considering the influence of chemical treatment on the mechanical performance of SPF-reinforced polymer composites. Thus, this review aims to comprehensively gather and discuss recent works accomplished on the influence of various chemical treatments on the mechanical properties of SPF-reinforced polymer composites. At the end of the manuscript, specific discussions are presented on current usage and potential applications of SPF-reinforced polymer composites to replace synthetic materials (e.g., glass fibres). A general overview of the comparison of natural (lignocellulosic) fibres and synthetic fibres is shown in Table 1. It is expected that this review article can encourage researchers and manufacturers to make progress in the development of SPF-reinforced polymer composites for various engineering applications to reduce carbon emissions.

## 2. Sugar Palm (*Arenga pinnata*)

Sugar palm is a *Palmae* tree family scientifically known as *A. pinnata*. The *Palmae* family has about 181 genera and more than 2600 species worldwide [60]. Currently, there are abundant lignocellulosic fibres of sugar palm. This tree is commonly found in tropical climate regions, including the South and Southeast Asian tropics. Geographically, the plant distribution includes the Indo-Malay archipelago, which includes Southeast Asian nations, such as the Philippines, Indonesia, and Malaysia [61]. Sugar palm is considered one of the most economical plants in Asia. A sugar palm tree is tall and has long-shaped leaves with decorative trunk fibres. Recently, a new way to harvest sugar palm by-products efficiently has been developed. The first sugar palm factory has recently been constructed in Tomohon, Indonesia [62]. The by-products of sugar palm include sugar, sago, bioethanol, alcohol, fibre, and thatch. The bioethanol from sugar palm can be used as an alternative fuel because the tree has higher alcohol production capability compared to sugarcane, sweet sorghum, and cassava [63,64]. Besides, SPF is considered a primary by-product of sugar palm. The fibres can be used in many applications, including brooms, septic tank base filters, and ropes for sea cordage, and in certain parts of Indonesia, the fibres are used as paintbrushes, clear water filters, and carpets [50].

## 3. SPF

**Figure 1:**
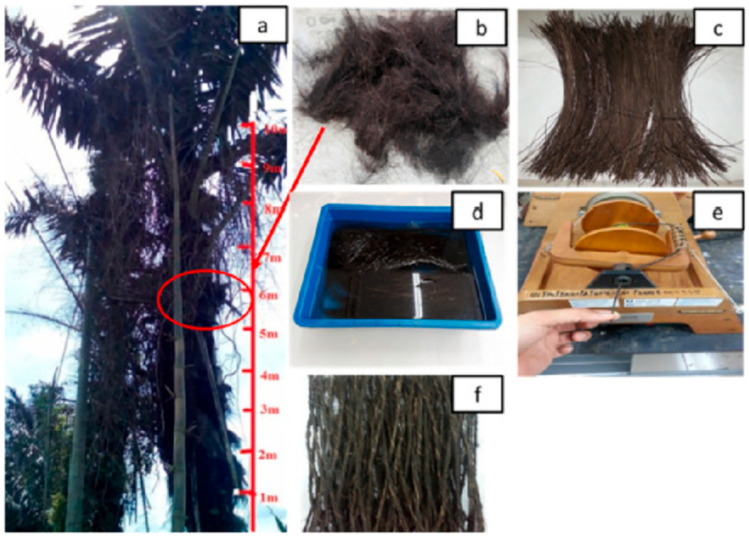
Preparation of SPF from sugar palm tree: (**a**) locating suitable SPF at the trunk, (**b**) SPF bundle, (**c**) combed SPF, (**d**) SPF treatment by alkalisation, (**e**) fibre yarning, and (**f**) finalised SPF yarn. Adapted with permission from Ref. [71]. Copyright Elsevier.

Sugar palm fibre is black fibre that can be found naturally in Malaysian and Indonesian rainforests [65]. Recently, SPF has received growing demands from consumers as the fibre can be used in various applications, such as ropes, roofs, brushes, brooms, pultruded components, mats, and hammocks [66]. The fibre can be harvested after five years, when the mature fibre is black with an approximate length of 1.19 m [53,67]. *Ijuk* or *Injuk* is the black fibrous hair covering the sugar palm trunk [68]. Sugar palm fibre is generally considered a waste product from sugar palm cultivation. This fibre also has considerable potential as reinforcement for biocomposite products. With high cellulose content, many researchers have studied the utilisation of SPF due to its high potential in industrial applications, such as photovoltaic backsheet [69], packaging products [40], and automotive components [70]. Figure 1 shows the extraction of SPF from sugar palm trunk.

### 3.1. Physical and Mechanical Characteristics of SPF

SPF can have a length of up to 1.19 m with a diameter of approximately 94–370 µm, as described by Bachtiar et al. [72]. The overall density of SPF is about 1.26 kg/m^3^, and the strength of the fibre depends on the maturity and altitude of the fibre harvested from sugar palm tree [73]. SPF is heat-resistant until 150 °C, with a flash point of 200 °C [64]. The harvested SPF from its tree trunk is segregated based on its grade from A to E, depending on its dimensions (thickness and length), as stated by Ishak et al. [53]. Bachtiar et al. [74] reported that the tensile strength, tensile modulus, and elongation at break of SPF are 190.29 MPa, 3.69 GPa, and 19.6%, respectively.

According to Nurazzi et al. [75], SPF consists of black fibre with high tensile strength that is comparable to coir, bamboo, and kenaf fibres. Table 2 presents the comparison of the mechanical behaviour of SPF with other lignocellulosic fibres. The main advantage of SPF is that the fibre is durable with a long life, as it is unaffected by heat and moisture compared to coir fibre. SPF is resilient to seawater; hence, the fibre can be embedded in marine applications [25]. Concerning the application of SPF as reinforcement in polymer matrix composites, various studies have been conducted on the properties of SPF-reinforced polymer composites [76,77,78].

### 3.2. Chemical Composition of SPF

The chemical compositions of lignocellulosic fibres are highly influenced by the type and nature of fibres. Thus, the fibres exhibit different characteristics. The properties of each component in sugar palm tree have a significant influence on the overall properties of SPF. The chemical compositions of lignocellulosic fibres depend on maturity, the tree part where the fibre is harvested, locality, and climatic conditions [51,73]. Sugar palm fibre is mainly made up of cellulose (α-cellulose), lignin, pectin, hemicellulose, and waxes. Hemicellulose acts as a compatibiliser between lignin and cellulose components [40,79]. Table 3 lists the chemical composition of SPFs from various tree parts. Cellulose provides strength and stability to cell walls to maintain the structural integrity of the fibres. Based on the aforementioned table, it can be seen that frond fibre has the highest cellulose content. However, SPF is usually obtained from *ijuk* part as it consists of almost 90% of the total fibre from a sugar palm tree.

In addition, the chemical composition of SPF also depends on the height of the tree where the fibre is harvested, as shown in Table 4. According to Ishak et al. [80], the chemical composition of SPF (i.e., cellulose, lignin, and hemicellulose) increased as the height of sugar palm tree increased. Nevertheless, these contents are significantly reduced as the tree becomes older. The SPF from the bottom part of the tree consists of high impurities, including silica. This bottom part of SPF indicates higher ash content (30.92%) in comparison to the fibre obtained from the upper part of the tree (2.06–5.84%) based on FTIR analysis. Owing to its remarkably high ash content, the fibre from 1 m height indicated lower moisture content (5.36%) than other fibres at 3–15 m height in the range of 7.72–8.7%.

## 4. Limitation of Lignocellulosic SPF as Reinforcement Material in Polymer Composites

SPF is lignocellulosic fibre made up of lower cellulose content of approximately 37.3–66.48%. Cellulose content gives lignocellulosic reinforcement material remarkable mechanical properties because cellulose acts as a building block for fibre. For instance, Mukhtar et al. [50] determined that flax fibre has higher tensile strength and modulus than SPF due to the higher cellulose content of flax fibre. Additionally, Dai [81] mentioned that flax fibre is made of lignin matrix that lies parallel to the fibre axis. Based on the discussion, cellulose acts as a vital structural component in lignocellulosic fibre that provides good durability and better structural integrity; hence, the drawback of SPF needs to be overcome.

As SPF is classified as lignocellulosic fibre, lack of interfacial bonding is considered as the main drawback of reinforcement material in composites. There are four interfacial bonding issues, such as interdiffusion bonding, mechanical interlocking, electrostatic bonding, and chemical bonding. The lack of SPF compatibility with the polymer matrix results in the low mechanical performance of final composites as there is no interaction with SPF that contains polar moieties. The non-polar nature of SPF usually influences the low dispersion of the fibre. Subsequently, the SPF experiences agglomeration within the polymer matrix due to the hydrogen bonds within the hydroxyl group, resulting in poor fibre dispersion within the matrix and poor matrix–fibre interaction. The non-polar hydrophobic nature of the polymer matrix impairs the dispersity of SPF, which is hydrophilic by nature. This phenomenon also leads to low interfacial adhesion, poor resistance to moisture uptake, and low melting point, which could initiate composite microcracks [39,82]. The effectiveness of the composites reinforced with lignocellulosic fibres relies on the fibre–polymer matrix interface and the tendency of transferable stress to the fibres from the matrix.

The lack of compatibility of lignocellulosic fibre/matrix can reduce the reactive area to bind the composites [83]. Consequently, this leads to crack propagation in composite laminate due to the occurrence of air voids between the fibre interface and the matrix [84]. Therefore, this phenomenon reduces the impact and tensile properties of the composites [85,86]. In this case, a comprehensive step needs to be taken, including fibre treatment (e.g., chemical modification) to remove impurities and unwanted components. Subsequently, the mechanical performance of SPF-reinforced polymer composites can be improved.

## 5. Fibre Treatments on SPF: Chemical Modification

Chemical pretreatment of SPF can enhance the morphological properties of the fibre, leading to high tensile and compressive strength. This is due to the enhancement of interfacial linkage of fibre–matrix adhesion [10]. In this point of view, the main contributor of strength and toughness in lignocellulosic fibre reinforced polymer composites is fibre–matrix adhesion, and efficient stress distribution of fibre–matrix determines the brittleness and toughness of composites [87,88]. Chemical treatment techniques for lignocellulosic fibres help clean the fibre surface, increase the surface roughness of fibres, and reduce the moisture absorption process [89]. The modification of lignocellulosic fibre surface can be performed by various treatments [90,91,92,93], such as gamma or electron beam irradiation [94,95], seawater [96], UV irradiation [97], plasma [98,99], water retted [100,101,102,103], corona [104,105], mercerisation [60,106,107,108,109,110,111,112,113,114,115,116,117,118,119,120,121,122], hydrothermal [123], and impregnation and chemical coupling [124,125]. For chemical modification of SPF surface, the treatments include benzoylation, peroxide treatment, peroxide treatment, graft copolymerisation, etherification, acetylation, permanganate treatment, mercerisation, and application of coupling agents (e.g., silane) [126]. For chemical treatment of fibre, an optimum fibre volume in the polymer matrix [127,128] and also manufacturing and processing techniques [30] allow remarkable improvement in composite strength and stiffness due to a high aspect ratio that effectively transfers stress to the matrix.

### 5.1. Alkalisation

Alkali treatment, also known as alkalisation, utilises various concentrations of sodium hydroxide (NaOH) solution to remove impurities in lignocellulosic fibres, hence elevating the fibre compatibility in matrices [129,130]. In this chemical treatment, which is well-known as mercerisation, untreated lignocellulosic fibre is immersed in NaOH solution for a certain period [131,132,133]. One of the advantages of alkalisation is the interruption of hydrogen bonds in the chemical structure, which creates a rough fibre surface. The immersion of lignocellulosic fibre in NaOH solution allows the removal of certain plant cellular components, such as hemicellulose, wax, lignin, and oils. These components inhibit the fibre from being reinforced in the polymeric resin [134]. The treatment allows the fibre to be fibrillated into fibre bundles, which shortens the fibre diameter, increases the aspect ratio, and influences the crystalline properties. Subsequently, the treatment converts cellulose I into cellulose II, as mentioned by Liu et al. [135]. Mercerisation allows the ionisation of the hydroxyl group to alkoxide, as shown in the following equation:

(1) $$\mathrm{Fibre}-\mathrm{OH}+\mathrm{NaOH}\;\to \mathrm{Fibre}-{\mathrm{ONa}}^{+}+H_{2}O$$

From Equation (1), it can be determined that the immersion of SPF in NaOH solution results in high swelling. A new Na–cellulose lattice is formed after the removal of surplus NaOH, which is a lattice with relatively large distances between cellulose molecules, and these spaces are filled with water molecules. At that moment, the OH groups of cellulose are converted into ONa-groups (Equation (1)), expanding the molecules’ dimensions. Later, rinsing with water will remove the linked Na ions and convert the cellulose to a new crystalline structure, which is considered a treated cellulose structure [136]. Expanding the molecules’ dimensions means that the number of reaction sites increases, and therefore, better adhesion between the fibre and the matrix. Hosur et al. [137] found that lignocellulosic fibre (e.g., flax) treated with 2.5 wt% alkaline solution for 1 h showed the highest degree of crystallinity, which resulted in the synergistic effect on flexural and water absorption performance.

Bachtiar et al. [138] discovered that overtreatment of SPF with mercerisation deteriorated the fibre. This statement is supported by Ticoalu et al. [139], in which changes in the morphology of fibre and reduction of tylose content on the surface of lignocellulosic fibre. Table 5 reviews studies in the literature on alkali treatment of SPF in order to improve fibre morphology and enhance the overall mechanical performance of biocomposites.

### 5.2. Silane Treatment

Chemical treatment using coupling agents, such as silane (SiH_4_), is another pretreatment method to increase fibre/matrix compatibility. This method stabilises biocomposites by reducing the number of hydroxyl groups in cellulose. Alkoxy silanes can form hydroxyl group bonds. Coupling agents (e.g., triethoxyvinyl silane and toluene diisocyanate) have been utilised in natural fibre treatment to enhance the interphase properties of natural fibre and polymer matrix. Hydrolysis, condensation, and formation of bonding occur in silanes. Silanols can form polysiloxane structures by reacting with the hydroxyl group of fibres. In the presence of moisture, the hydrolysable alkoxy group leads to the formation of silanols. The chemical reaction formula can be deduced from the silane reaction:

(2) $$\mathrm{Fibre}-\mathrm{OH}+R-\mathrm{Si}{\left(\mathrm{OH}\right)}_{3}\to \mathrm{Fibre}-O-\mathrm{Si}{\left(\mathrm{OH}\right)}_{2}-R$$

Subsequently, the silanol reacts with the hydroxyl group in SPF to covalently bond to the cell wall. This phenomenon confines hydrocarbon chains from the swelling of the fibre, producing an intertwined network from the diffusion of hydrocarbon chains with polymer matrices [142,143]. Additionally, this silane treatment aids in the restructuring of hydrocarbon chain, which can influence fibre wettability [144]. Thus, silane pretreatment enhances the chemical affinity of the matrix, which consequently improves the tensile strength of the final biocomposite by reducing the effect of moisture [92,145].

**Figure 2:**
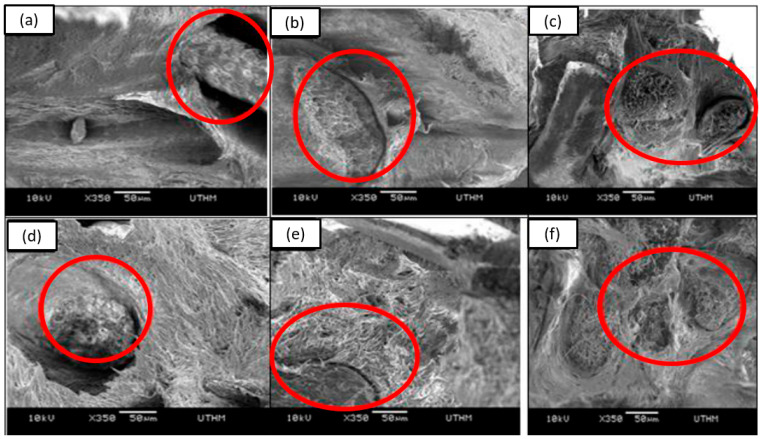
SEM analysis of fractured SPF-reinforced propylene composites with (**a**) 10 wt%, (**b**) 20 wt%, (**c**) 30 wt% of untreated SPF; (**d**) 10 wt%, (**e**) 20 wt%, (**f**) 30 wt% of silane treated SPF. Adapted with permission from Ref. [148]. Copyright Elsevier.

According to Atiqah et al. [146], silane treatment could separate lignin and hemicellulose from the lignocellulosic SPF as the fibres were agitated for 3 h in 2% silane solution. Moreover, the silane treatment on SPF greatly reduced the number of cellulose hydroxyl groups in SPF as compared to the mercerisation of SPF. The research also discovered that this treatment enhanced the degree of crosslinking and increased the active bonding surface area of SPF. This finding is aligned with the work performed by Huda et al. [147]. In another study by Zahari et al. [148], the scanning electron microscopy (SEM) analysis found that narrower gaps were observed at the fracture sites of silane-treated SPF-reinforced polymer composites, as shown in Figure 2. They concluded that silane treatment provides better surface compatibility of SPF.

### 5.3. Sodium Bicarbonate Treatment

Sodium bicarbonate treatment is another notable chemical modification of SPF, in accordance with Mukhtar et al. [23]. The researchers treated SPF with 10% sodium bicarbonate solution by soaking the fibre for 5 days. For this chemical modification, SPF was washed thoroughly from excess sodium bicarbonate and the fibres were ensured to have neutral pH prior to drying for 24 h in an oven. The authors discovered that the percentage improvement of SPF due to sodium bicarbonate treatment was relatively lower than alkalisation. From the SEM analysis, it was revealed that the removal of waxy layers on SPF was lower using this treatment.

### 5.4. Benzoylation

**Figure 3:**
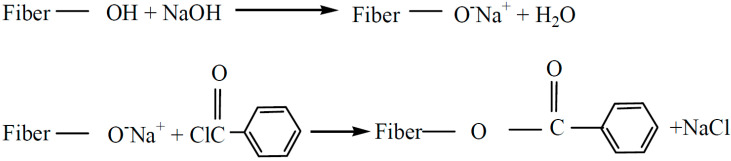
A possible reaction between cellulosic-OH groups and benzoyl chloride. Adapted from Ref. [152]. Creative Common CC BY license.

Benzoylation is a useful chemical treatment of SPF as it can enhance the tensile, thermal, and morphological properties of the fibre [149]. In common practice, benzoyl treatment took place when SPF was pre-soaked for 30 min in 18% NaOH solution before being washed and dried. After that, the fibres were suspended in 10% NaOH solution and agitated with 50 mL benzoyl, based on the experiment conducted by Safri et al. [150]. According to Vijay et al. [151], the treatment of the lignocellulosic fibre from *Ipomoea pes-caprae* significantly improved the fibre surface roughness, providing more activation areas for combination with polymer matrices. This resulted in the enhancement of fibre wettability in the matrix, thus improving the flexural and tensile performance of composites. Figure 3 shows the reaction between the cellulosic –OH group of natural fibre and benzoyl chloride.

### 5.5. Other Fibre Treatment

**Figure 4:**
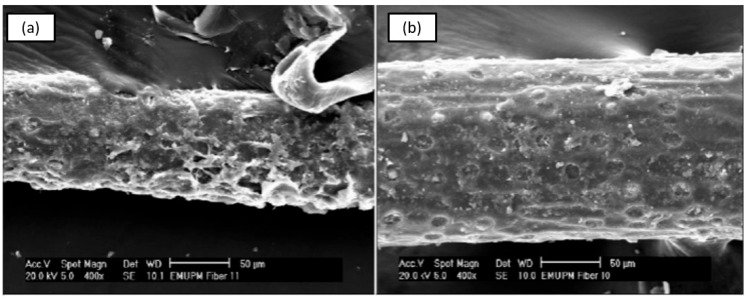
SEM analysis of (**a**) untreated and (**b**) seawater treated SPF. Adapted with permission from Ref. [153]. Creative Common CC BY license.

Fibre treatment of SPF can also be conducted via seawater treatment, which is considered a low-cost and efficient pretreatment to improve fibre surface topography. For SPF, many researchers have studied seawater treatment, where the fibre is con-ventionally applied in long-term seawater exposure, in accordance with Sanyang et al. [52]. The treatment is usually conducted using SPF immersed in seawater (0.035% salt for 1 L water) for several days. Hence, this treatment indicates that it is potentially useful to enhance the properties of SPF at lower costs. Ishak et al. [153] investigated the influence of seawater treatment on the properties of SPF. They conducted the ex-periment by immersing SPF for 30 days prior to testing. A significant improvement in the surface topography of SPF was observed, as indicated in Figure 4. They deduced that the seawater treatment improved the surface properties of SPF by eliminating the outer layers of hemicellulose and pectin, which subsequently enhanced the fibre rein-forcement potential.

### 5.6. Combined Chemical Treatments

A combination of chemical treatments has been experimented on SPF, such as alkalisation-silane treatment. According to Atiqah et al. [146], they evaluated the bonding strength of combined alkali-silane treatment of SPF-reinforced thermoplastic polyurethane (TPU) composites. In this work, SPFs were soaked in a combined solution containing 6% NaOH and 2% silane solutions for 3 h. The SEM analysis of combined alkali-silane-treated SPF showed less impurities and rougher surface topography. Although the combined effect of alkali-silane treatment was estimated to produce remarkable changes in the fibre morphology, the research deduced that the combined treatment displayed only a slight improvement in the surface topography of SPF. The interfacial bonding strength decreased after the combined treatment, while the tensile strength improved slightly.

## 6. Previous Literatures on the Influence of Chemical Treatments on Mechanical Properties of SPF-Reinforced Polymer Composites

Various studies have been carried out to evaluate the potential of SPF under the influence of chemical treatment and the remarkable improvement of the properties of the fibre and its composites. The SEM results depict that treated fibre aids in removing the outer layers that contain impurities, such as ash, wax, and pectin. Researchers have also established significant enhancement in the tensile, flexural, and impact strength and stiffness of SPF-reinforced polymer composites [149,154]. Among chemical treatments of SPF include alkalisation, silane treatment, seawater treatment, sodium bicarbonate treatment, and combined treatments. Table 6 summarises the experimental results of recent works on chemically-modified SPF-reinforced polymer composites in terms of flexural, tensile, and impact properties.

### 6.1. Alkalisation

Based on previous literature, alkali treatment of SPF shows remarkable enhancement of the mechanical properties of SPF-reinforced polymer composites. According to Bachtiar et al. [72,155], the alkali-treated SPF-reinforced epoxy composites revealed an outstanding increase in flexural and impact strength of approximately 24.41% and 12.85%, respectively, compared to untreated SPF-composites. Bachtiar et al. [155] discovered a significant impact of alkali treatment of SPF on the composite properties via the hand lay-up method. Based on their findings, the impact strength of SPF-reinforced epoxy composites increased by 28.69% via 0.5 M NaOH treatment of SPF for 8 h. They deduced that a rougher surface on the fibre interface due to chemical treatment created many activation areas to increase the compatibility with the polymer matrix. This condition allows better permeability and inhibited detachment, debonding, or pull-out of SPFs [159,160].

Atiqah et al. [161] and Mohamed et al. [162] discovered that the alkali treatment at an optimum value of 6% concentration resulted in significant results in the mechanical properties of SPF-reinforced thermoplastic polyurethane composites. Additionally, the tensile strength of SPF improved under 6% NaOH treatment with optimum immersion time [163,164]. From these studies, it can be deduced that fibre treatment at 6% NaOH is good for splitting SPF bundles into very fine fibres. Subsequently, this phenomenon results in effective entrance of resin and triggers high intertwining of fibres in the matrix [165]. Subsequently, it leads to better interfacial adhesion with enhanced fibre/matrix bonding.

Chalid et al. [156] determined that the tensile strength and elastic modulus of SPF-reinforced polylactic acid (PLA) increased as 20% fibre content was treated using 0.25 M NaOH solution for 30 min. They concluded that the alteration of the SPF interface strengthened the mechanical interlocking of SPF fibrils with PLA resin. Moreover, trapped voids and fibre pull-out can be reduced by stirring during mixing the fibre with dissolved PLA resin under the effect of alkali treatment [166,167].

### 6.2. Silane Treatment

Atiqah et al. [146] evaluated the influence of alkali, silane, and combined treatment on SPF-reinforced polyurethane composites. They revealed that 2% silane-treated SPF exhibited better tensile strength than those of 6% alkali-treated, alkali-silane-treated, and untreated SPF composites. Specifically, the silane-treated SPF composite exhibited 17.64% higher tensile strength than the untreated SPF-composite. Meanwhile, the interfacial stress strength of the silane-treated SPF-composite was higher than alkali- and combined-treated SPF-composites. The micro-surface of the silane-treated SPF composite was roughened to induce mechanical interlocking with the polyurethane matrix.

Zahari et al. [148] established that silane-treated SPF/polypropylene (PP) composites elevated the elastic modulus and tensile strength up to 1.098 GPa and 23 MPa, respectively. In this case, they implemented 30 wt. % SPF, which was an optimum fibre loading, and treated the fibre with 2% vinyltrimethoxysilane for 15 min to enhance the adhesion of the fibre with the polymer matrix. Based on the morphological analysis, the untreated SPF/PP composites indicated poor interfacial adhesion due to the obvious gaps between the fibre and the matrix. However, the gaps were significantly less noticeable and became narrower as the SPF was treated with a silane coupling agent. Therefore, it can be deduced that SPF and thermoplastic matrix have better compatibility when treated with silane solution. Moreover, the optimum SPF content along with silane treatment enhanced the stiffness of composites. The treatment allows better distribution of SPF within the PP matrix [168].

### 6.3. Sodium Bicarbonate Treatment

Researchers have also used sodium bicarbonate for SPF treatment as an effort to enhance SPF-reinforced polymer composites. Mukhtar et al. [23] prepared a pretreatment solution with 10 wt% bicarbonate to modify the SPF. The treatment was conducted with 5 days of immersion and later, the fibre was washed completely with distilled water to remove excess treatment solution. Later on, Mukhtar et al. [7] conducted an experiment to evaluate the influence of the concentration of sodium bicarbonate on the tensile properties of SPF/PP composites. The improvement in tensile performance was observed due to the sodium bicarbonate-treated SPF that removed excess impurities on the fibre, and the treated SPF composite recorded 58.76 MPa against 53.01 MPa for the untreated SPF composite.

### 6.4. Benzoylation Treatment

Safri et al. [157] conducted a study of benzoylation treatment to modify SPF and also to reinforce epoxy composites. They determined that this treatment mechanism improved the interfacial adhesion of SPF–reinforced epoxy composites. Major improvement in the tensile properties of the SPF/epoxy composites was observed upon the benzoyl treatment of SPF. The rougher surface of the treated SPF after benzoylation treatment provides more activation areas for combination with polymer matrices. This condition enhances fibre wettability, which is good for the mechanical properties of SPF-reinforced polymer composites.

### 6.5. Seawater Treatments

Seawater treatment is another fibre treatment commonly used by researchers to enhance SPF-composites. Ishak et al. [153] discovered that the impact and flexural strength of 30 wt% SPF-reinforced epoxy composites increased significantly by 5.06% and 7.35%, respectively. This study established that seawater treatment with 30 days of fibre immersion improved the surface properties of SPF by removing the outer layers of hemicellulose and pectin. Subsequently, this leads to improved interfacial bonding between the fibre and the matrix.

Maisara et al. [96] assessed the effect of fibre length and seawater treatment on the mechanical properties of SPF-reinforced unsaturated polyester composites. They found out that 30 wt% SPF with 15 cm length treated with seawater exhibited significant tensile and flexural strength of 18.33 and 80.80 MPa, respectively. However, this type of SPF/unsaturated polyester composites exhibited the lowest tensile modulus of 4251.96 MPa. They concluded that the removal of impurities (i.e., pectin and waxy substances) from the SPF surface and the creation of a rougher surface after seawater treatment promoted mechanical interlocking for better mechanical properties of SPF composites. However, the effect of seawater treatment degraded the structural integrity of fibre, which subsequently decreased due to the elasticity of SPF and its composites.

### 6.6. Combined Chemical Treatments

Atiqah et al. [146] evaluated the influence of combined silane–alkali treatment on SPF-reinforced polyurethane composites. Although the combined treatment was expected to improve SPF surface, the treatment resulted in less impact compared to a single treatment. For instance, the tensile strength and modulus of the combined-treated SPF-reinforced polyurethane composites were lower than alkali- and silane-treated SPF-composites. This is because the mixture of both chemicals does not have any effect on SPF [169]. Furthermore, the combined effect of silane and alkali degraded the structural integrity of SPF, as cellulose was washed away by both treatment reactions.

Another combined treatment conducted by Mohammed et al. [158] implemented microwave and alkaline treatments on SPF-reinforced polyurethane composites. This offers excellent tensile properties compared to the use of alkaline-treated and untreated SPF/polyurethane composites. Based on their findings, microwave treatment at 70 °C assisted the fibre treatment process by removing impurities at the outer layer of the fibre after alkali treatment. Additionally, the microwave treatment reduced excess moisture in SPF, which in turn increased the interfacial adhesion of fibre/matrix [170]. Nevertheless, this combined treatment does not significantly improve the overall strength of SPF composites because higher temperatures may lead to fibre damage, consequently reducing the reactive sites for mechanical interlocking.

## 7. Applications of SPF-Reinforced Polymer Composites

Currently, issues related to environmental pollution have become increasingly prominent among global communities due to global warming and haze [171,172]. Many strategies and efforts have been aligned by conducting new research and developing new technologies to reduce the effect of these catastrophic events. Thus, many material scientists and engineers have utilised agricultural wastes as a substitute for current synthetic materials as these wastes are renewable resources. Sugar palm fibre is seen as an emerging natural-based material that can fulfil the needs to mitigate the current issue due to its high mechanical performance and lightweight in engineering applications. These benefits enable SPF to compete with the majority of natural fibres on the market, including coir, kenaf, cotton, and jute due to its improved mechanical characteristics through fibre treatment [88,173]. For instance, SPF-reinforced epoxy composites have been implemented to develop a safety helmet. This is because SPF exhibits good water resistance and can endure high impact forces [174]. The SPF/epoxy helmet is referred to as Helmet-Ijuk Reinforced Composite (HIReC) [175].

**Figure 5:**
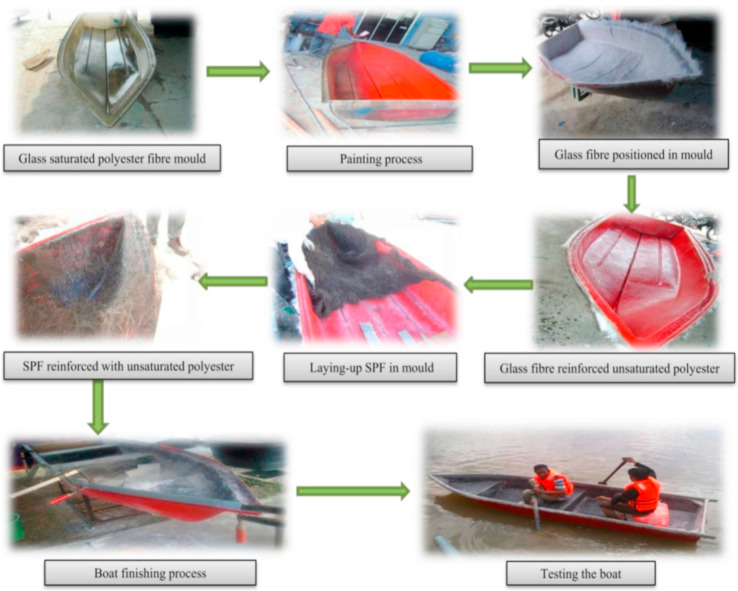
Fabrication of SPF/glass fibre reinforced UPE composites fishing boat. Adapted with permission from Ref. [52]. Copyright Elsevier.

Sugar palm fibre is well-known due to its high durability in seawater. The fibre exhibits good resistance to seawater; hence, the fibre is usually applied as shipping ropes [52,176]. Figure 5 displays the fabrication process of a natural fibre composite boat made from SPF and glass fibre-reinforced unsaturated polyester (UPE) composites [25,72]. The SPF/glass fibre/UPE composite boat was manufactured using compression moulding and the hand-layup technique. The weight of the SPF-composite boat declined by approximately 50% as SPF was used to replace a certain amount of glass fibre. The density of SPF is 1.22–1.26 kg/m^3^, which is approximately half of the density of E-glass fibre (2.55 kg/m^3^) that is commonly used in manufacturing fishing boats.

SPF possesses outstanding mechanical performance, where the fibre becomes a promising candidate as a reinforcement material in the polymer matrix. Recently, many developments in design have been executed to develop automotive parts from SPF-reinforced polymer composites, including automobile engine mounting [27], side door impact bar [177], parking lever brake [178], antiroll bar [179], and automotive bumper [180]. Other than automotive applications, SPF is also highly potential for construction applications, as it is composed of lignin, in accordance with Jędrzejczak et al. [181]. In their project, they recommended deriving lignin from lignocellulosic fibre, such as SPF, to harvest and implement sustainable epoxy and phenol-formaldehyde resins for construction usage. There are many potential applications of SPF as nanocellulose by reinforcing the fibre in starch-based composites for packaging applications [20,119].

## 8. Conclusions and Future Outlook

This article focuses on previous literature related to the chemical treatment of SPF and the mechanical properties of SPF-reinforced polymer composites. Sugar palm fibre has notable mechanical performance, such as flexural, tensile, and impact strength. Different tests have been applied to determine the strength and stiffness in impact, tension, and bending mode. The fibre shows good hydrophilic properties, elevating the water absorption capacity of the fibre. This property inhibits the wettability of lignocellulosic fibre with the polymer matrix to form good compatibility fibre/matrix for high-strength composites. The drawback of SPF is also influenced by inhibitors, such as wax and pectin, which further reduce the interfacial adhesion between the fibre and the matrix. Several researchers have suggested the use of chemical treatments on SPF to resolve this issue, such as alkalisation, silane treatment, sodium bicarbonate treatment, benzoylation, and seawater treatment. Several studies have also proposed combined treatments and evaluated the effectiveness of the treated SPF-reinforced polymer composites. Based on previous literature, silane treatment of SPF with 2% concentration for 3 h significantly improved the fibre compatibility with thermoplastic matrix composites. Additionally, combined treatments, such as silane–alkali and alkali–microwave treatments, are ineffective in improving the fibre topography and wettability to be reinforced in the polymer matrix as compared to a single treatment. This is due to the double actions from two distinct treatments, which can degrade fibre structure. Overall, silane-treated SPF-reinforced polyurethane composites demonstrate superior mechanical properties to other treated SPF-reinforced polymer composites. It can be seen that treated SPFs have high potential to substitute synthetic fibres (glass fibres) for tensional, bending, and impact applications. Subsequently, the application of SPF as fibre reinforcement of polymer composites shows significant possibility for the automotive, locomotive, construction, and housing sectors.

At the end of the review, it can be established that treated SPF-reinforced polymer composites can be used in various sectors, especially in high impact equipment, such as helmets, antiroll bars, and anticrash boxes. Additionally, seawater-treated SPF-reinforced polymer composites are ideal to be implemented in fishing boat manufacturing because the composites have great resistance to seawater. Construction and building materials are the most interesting application field, given that SPF can improve the properties of wood, concrete, steel, and glass as the primary construction materials. For future work, it is suggested to apply treated SPF-reinforced polymer composites for construction and structural components, such as concrete beams, cross-arms in transmission towers, and wind turbine beams. Thus, SPF can potentially be a serious contender in advanced material applications.

## Figures and Tables

**Table 1:** Comparison of natural fibres with synthetic fibres [54,55,56,57,58,59].

Aspects	Synthetic Fibres	Natural Fibres
Examples	Glass fibres	Lignocellulosic fibre such as SPF
Density	High	Low
Biodegradability	Took long period for the material to be decomposed.	Highly biodegradable
Energy usage	Required huge amount of energy input during processing	Low energy consumption during its processing
Environmental effect	Huge negative impact toward environment if disposal is not handled properly	Environmentally friendly since it is extracted from bio-waste
Health effect	Serious issues toward respiratory diseases	No effect toward health
Recyclability	Cannot be recycled	Recyclable
Raw material cost	Relatively high price	Low price

**Table 2:** Comparison of the mechanical performance of SPF with other lignocellulosic fibres. The data are adapted from Ref. [37]. Creative Common CC BY license.

Fiber	Density (g/cm^3^)	Tensile Modulus (GPa)	Tensile Strength (MPa)	Elongation at Break (%)
Sugar Palm	1.292	4.96	156.96	7.98
Bagasse	1.5	17	290	-
Bamboo	1.25	11 to 17	140 to 230	-
Flax	0.6 to 1.1	27.6	345 to 1035	2.7 to 3.2
Hemp	1.48	70	690	1.6 to 4
Jute	1.3	26.5	393 to 773	1.5 to 1.8
Kenaf	1.45	53	215.4	1.6
Sisal	1.5	9.4 to 22	511 to 535	2.0 to 2.5
Pineapple	0.8 to 1.6	1.44	400 to 627	14.5
Coir	1.2	4 to 6	138.7	30

**Table 3:** Chemical compositions of SPF from different tree parts. Data extracted from Ref. [68]. Creative Common CC BY license.

Chemical Composition (%)	Sugar Palm Tree Parts			
	*Ijuk*	Trunk	Bunch	Frond
Lignin	31.5	46.4	23.5	18.9
Hemicellulose	65.6	61.1	71.8	81.2
Cellulose	52.3	40.6	61.8	66.5
Extractive	4.4	6.3	2.2	2.5
Moisture	7.4	1.5	2.7	2.7
Ash	4.0	2.4	3.4	3.1

**Table 4:** Chemical composition of SPF obtained from various heights of the tree. Data are adapted from Ref. [53]. Copyright Elsevier.

Chemical Composition (%)	Height (m)							
	15 (Top)	13	11	9	7	5	3	1 (Bottom)
Lignin	24.9	24.3	23.0	23.6	20.5	20.9	18.9	17.9
Hemicellulose	7.5	7.9	7.9	7.9	7.7	7.4	6.1	4.7
Cellulose	53.4	54.4	55.8	56.8	56.6	55.3	49.4	37.3
Extractive	1.0	1.2	1.5	1.4	1.4	1.7	2.0	2.5
Moisture	8.7	8.1	7.7	8.2	8.4	7.9	8.6	5.4
Ash	4.3	4.0	4.1	2.1	4.2	5.8	14.0	30.9

**Table 5:** Research on alkalisation of SPF with various concentrations and immersion times.

Concentration of NaOH Solution	Immersion Time (Hours)	References
4 and 6%	1	[140]
5 and 10%	2	[139]
6%	3	[141]
0.25 M and 0.5 M	1, 4, and 8	[138]

**Table 6:** Reported works on the mechanical properties of SPF-reinforced polymer composites.

Fibre Condition	Matrix	Matrix Type	Chemical Treatments	Details	Flexural		Tensile		Impact	Ref.
					Strength (MPa)	Modulus (GPa)	Strength (MPa)	Modulus (GPa)	Strength (kJ/m^2^)	
10 wt% of SPF (long fibre)	Epoxy	Thermo-set	Alkali	0.5 M of NaOH solution at 8 h	90.68	4672	41.88	3780	6.0	[72,138,155]
30 wt% of SPF (powder fibre)	Phenolic	Thermo-set	Alkali	0.5% of NaOH solution at 4 h	92.59	5.17	-	-	7.28	[18]
10 wt% of SPF (Short fibre)	PLA	Bio-polymer	Alkali	0.25% of NaOH solutions	-	-	32.5	0.263	-	[156]
30 wt% of SPF (powder fibre)	Poly-propylene	Thermo-plastics	Silane	2 wt% of silane solution for 3 h	-	-	23.00	1.096	-	[148]
SPF (long fibre)	Poly-urethane	Thermo-plastics	Silane	2 wt% of silane solution for 3 h	-	-	173.44	10.07	-	[146]
30 wt% of SPF (mat fibre)	Poly-propylene	Thermo-plastics	Sodium bicarbonate	10 wt% of sodium bicarbonate solution for 5 days	60	2.47	58.76	2.06	17.61	[7]
10 wt% of SPF (long fibre)	Epoxy	Thermo-set	Benzoy-lation	18% NaOH solution for 30 min, 10% NaOH + Benzoyl chloride solution	-	-	22.7	3.62	-	[157]
30 wt% of SPF (long fibre)	Epoxy	Thermo-plastics	Seawater	Seawater 30 days	54.22	-	-	-	18.46	[153]
30 wt% of SPF (long fibre–15 cm)	Unsatu-rated polyester	Thermo-set	Seawater	Seawater 30 days from Port Klang, Selangor, Malaysia	80.80	-	18.33	4.374	-	[96]
30 wt% of SPF (Short fibre)	Poly-urethane	Thermo-plastics	Combine	4% of NaOH solution and 70 °C microwave treatment	-	-	18.42	1.307	-	[158]
SPF (long fibre)	Poly-urethane	Thermo-plastics	Combine	6 wt% NaOH and 2 wt% of silane solutions for 3 h each	-	-	142.09	7.75	-	[146]

## Data Availability

All information and data are available within the articles.
